# Awareness, attitude, and acceptance of newborn screening for sickle cell disease among health workers and caregivers at primary healthcare centers in Gwagwalada Area Council, Federal Capital Territory, Abuja, Nigeria

**DOI:** 10.3389/fpubh.2024.1453727

**Published:** 2025-01-07

**Authors:** Isa Hezekiah A, Chisomaga Ifeanyi Oparaugo, Grace Doyin Ajetomobi, Ayomide Esther Fasina, Reuben Ikechukwu Chianumba, Obiageli Eunice Nnodu

**Affiliations:** ^1^Center of Excellence for Sickle Cell Disease Research and Training (CESRTA), University of Abuja, Abuja, Nigeria; ^2^Department of Hematology and Blood Transfusion, University of Abuja, Abuja, Nigeria

**Keywords:** awareness, attitude, newborn screening, sickle cell disease, care givers

## Abstract

**Introduction:**

Newborn Screening (NBS) is a public health program designed to identify and provide early interventions for infants with genetic disorders such as Sickle Cell Disease (SCD). Lack of awareness and unwillingness to participate in the NBS by caregivers and some healthcare workers are major contributing factors impeding NBS for SCD.

**Objective:**

To evaluate the level of awareness and acceptance of NBS for SCD and the determinant factors influencing caregivers in Gwagwalada Area Council of the Federal Capital Territory, Abuja, Nigeria.

**Methods:**

The study employed a descriptive, quantitative design using questionnaires administered to healthcare workers and caregivers at immunization and antenatal clinics of 10 selected Primary Healthcare Centers (PHC) in Gwagwalada Area Council of the Federal Capital Territory, Abuja, Nigeria.

**Result:**

A total of 357 participants, comprising 301 caregivers and 56 health care workers responded to the questionnaires. Among the caregivers, 171 (57.2%) were not aware of NBS for SCD. Two hundred and ninety (97%) expressed willingness to participate in the NBS. However, 32 (10.6%) indicated reluctance to accept positive results of SCD. The majority, 175 (59.1%) of the caregivers strongly agreed that NBS for SCD helps in the early detection and management of SCD, while 7 (2.4%) disagreed. Notably 272 (90.4%) of the caregivers had secondary and tertiary education. Among the healthcare workers, 39 (73.6%) were aware of NBS for SCD and 30 (73.2%) have recommended it to caregivers.

**Conclusion:**

This study revealed a low level of awareness of NBS for SCD among caregivers. However, there was a high level of acceptance among them. The level of awareness is high among healthcare workers. Education emerged as the major factor determining the knowledge and attitude of caregivers toward NBS for SCD.

## Introduction

Sickle cell disease (SCD) is the most common hematological genetic condition resulting from the inheritance of a sickle cell gene from one parent and another abnormal hemoglobin gene from the other parent leading to the development of abnormal sickle-shaped red blood cells with a variety of clinical manifestations (1). SCD is a life-long disorder associated with morbidity and mortality (2). It is estimated that 312,000 newborns were born with sickle cell anemia globally in 2010, with 230,000 being born in Sub-Saharan Africa accounting for 80 percent of the global sickle cell anemia population (3, 4). In high-income countries, the life expectancy of SCD patients has increased dramatically over the last 40 years, reaching 50 years, whereas in Sub-Saharan Africa, most children with SCD are likely to die before reaching the age of 5 (5, 6). It is expected that large-scale universal screening stands the chance of saving up to 9,806,000 newborns with sickle cell anemia globally, 85% of these newborns will be born in sub-Saharan Africa (7). Model estimates from the Nigeria National Demographic Survey showed that the national average under-5 mortality for children with SCD born between 2003 and 2013 was 490 per 1,000 live births (95% CI 270–700), 40 times higher (95% CI 2·1–60) than children with HbAA, with about 42% (95% CI 17–69) of national under-5 mortality attributable to excess mortality from SCD (5).

Newborn screening (NBS) is a public health program designed to identify and provide early treatment to infants with critical and genetic health problems that are not obvious at birth (8, 9). In high-prevalence areas, there is evidence of several benefits of universal NBS for SCD (10). In Jamaica, neonatal screening for SCD has led to improved outcomes in the affected babies, while in Brazil, newborn screening for SCD has resulted in about 300,000 babies being screened across 36 municipalities by the end of the year 2000 (11). The national neonatal screening policy for diagnosis and management of SCD in Nigeria is for universal newborn screening in PHCs using point of care tests. However, this policy has not been properly disseminate the public and even among health care workers and the policy has not been translated into action. Consequently NBS is only taking place as pilot projects in some parts of the country. Most people with SCD are identified when they present with symptoms and the diagnosis is confirmed by qualitative hemoglobin electrophoresis, which is most often available at the teaching hospitals (12). Early screening of infants for SCD allows for early initiation of prophylactic treatment, education of parents/guardians and comprehensive management leading to a reduction in mortality. Evidence of multiple benefits of universal newborn screening for SCD in high prevalence regions has been demonstrated (13).

Parents’ level of knowledge about the NBS test was consistently reported to be below average across cultures (14). Mothers frequently conveyed receiving inadequate or no information about the newborn screening program (15). Even among those who received information from healthcare providers, awareness about the specific aspects of newborn screening was insufficient (14). It is important for mothers to have adequate understanding and awareness of newborn screening. Current studies showed that mothers who expressed increased awareness and higher levels of knowledge about newborn screening program were more committed to having the tests performed for their newborns (15).

There is very little information available for mothers and caregivers on NBS for SCD, despite the increasing mortality rate of children with SCD (1). The level of acceptance of the newborn screening is relatively low among mothers and health workers.

The purpose of this survey is to evaluate the awareness, attitude, and acceptability of NBS among caregivers and healthcare workers in PHC in Gwagwalada Area Council of the Federal Capital Territory, Abuja, Nigeria. As well as identify the factors leading to poor awareness and acceptance of NBS.

## Methods

### Study design and participants

This descriptive cross-sectional study employed questionnaires administered through the Research Electronic Data Capture (REDCap) software. Socio-demographic data and responses on awareness, acceptance, and attitudes toward NBS for SCD were collected between May and July 2023. Caregivers (mothers, fathers, aunts, grandmothers, and guardians) and pregnant women attending immunization and antenatal clinics, and healthcare workers from 10 selected Primary Healthcare Centers (PHC) in Gwagwalada Area Council, FCT, Abuja, were surveyed. The Healthcare workers comprised community health officers (CHO), community health extension workers (CHEWs), nurses, medical laboratory technologists and technicians, and medical record officers providing care in these primary health centers.

### Study setting and sites

**Figure 1:**
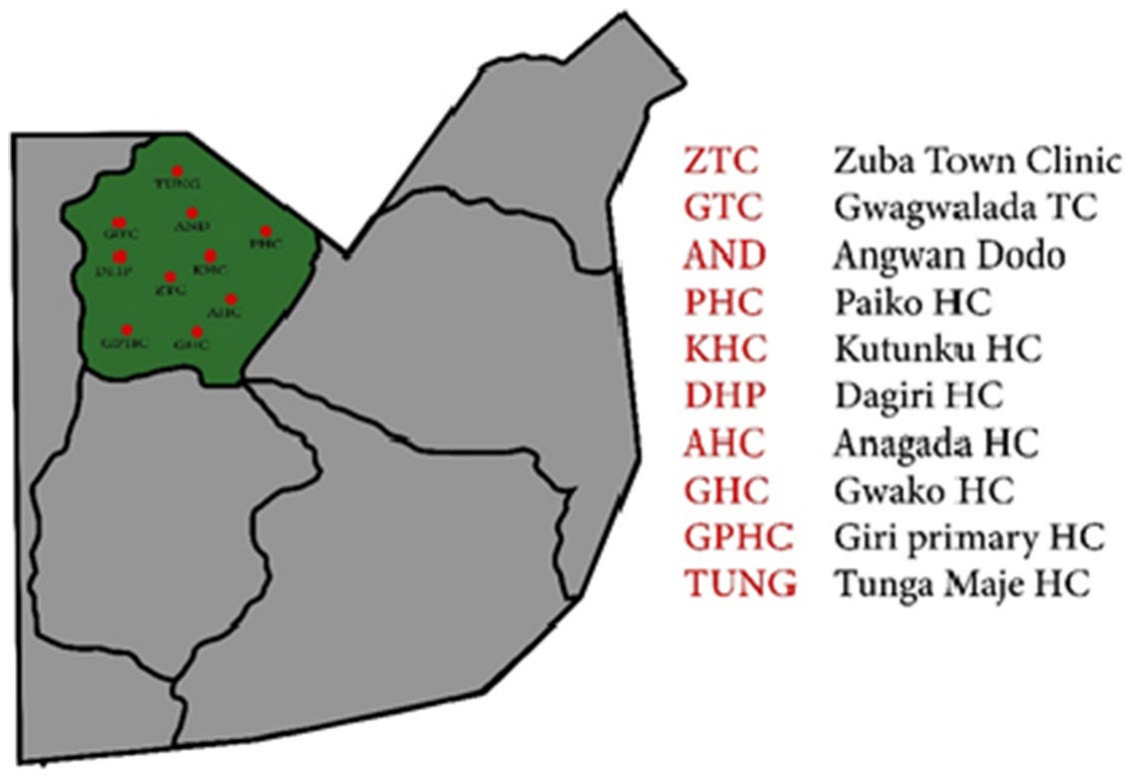
Map showing the locations of Primary Health Care Centers (red dots) within Gwagwalada Area Council, Federal Capital Territory.

The study was conducted in Gwagwalada Area Council situated in the Federal Capital Territory Abuja (16). Gwagwalada is geographically located at latitude 8°56′29″ North and longitude 7°5′31″ East. It is located about 59 Km away from the Central Business District of Abuja while the distance to Lagos, the former capital city of Nigeria is about 535 Km by air and 755 Km by road. Out of the 33 Primary Health Centers (PHCs) in the Area Council, 10 were selected for the study based on their strategic importance and accessibility to the population. The selected PHCs were Gwagwalada Town Clinic (GTC), Zuba Town Clinic (ZTC), Tungan Maje Primary Health Center (TUNG), Anagada Primary Health Center (AHC), Gwako Health Center (GHC), Giri Primary Health Center (GPHC), Kutunku Primary Health Center (KHC), Dagiri Primary Health Center (DHC), Agwan Dodo Primary Health Center, and Paiko Primary Health Center. See Figure 1.

### Justification for PHC selection

The 10 centers were chosen based on their strategic importance and accessibility to a large proportion of the Gwagwalada Area Council’s population. These centers have high patient turnout, and cover a wide geographical area within the council and comprise diverse communities. Additionally, the selected PHCs possess the necessary infrastructure and staffing to support the study’s logistical and operational requirements. The selecting these centers ensured a broader reach and a representative sample.

### Sample size calculation

For this study, we calculated the sample size required to achieve a 95% confidence interval with a 5% margin of error. The calculations were based on the population of the Gwagwalada Area Council, which is projected at 346,000 by the Nigeria Bureau of Statistics.

#### Caregivers

To determine the sample size for the study, we used the following formula for sample size calculation:


$$n=\frac{Z^{2}\cdot p\cdot \left(1-p\right)}{e^{2}}$$

Where:

n is the sample size.

*Z* is the *Z*-value [the number of standard deviations from the mean corresponding to the desired confidence level; for a 95% confidence interval, (Z = 1.96)].

p is the estimated population proportion (assumed to be 50%, or 0.5, to ensure maximum variability).

e is the margin of error (set at 5%, or 0.05).

Since our population size (*N*) is finite (346,000), we adjust the sample size using the finite population correction formula:


$$n\;\mathrm{adjusted}=\frac{n}{1+\frac{n-1}{N}}$$

Thus, the final sample sizes for this study are 330 caregivers and 60 healthcare providers. These sizes were chosen to ensure statistical significance and practical feasibility in representing the population of the Gwagwalada Area Council, healthcare providers at the sites and the number of caregivers attending immunization at the centers. However, only 301 caregivers and 56 healthcare providers completed the survey, others withdrew consent or did not complete the survey therefore were not added to the report.

### Ethical approval

Ethical approval was obtained by the Federal Capital Territory Health Research Ethics Committee (FHREC/2020/01/85/01-09-20). Written informed consent to participate in this study was provided to the participants.

### Inclusion criteria

Caregivers or women who were attending immunization or antenatal clinics and consented to participate in the study.

Healthcare workers who work in PHC in Gwagwalada Area Council and consented to participate in the study.

Caregivers and healthcare workers who were willing to respond to the questionnaire.

### Exclusion criteria

Care givers or women who were attending immunization or antenatal clinics but were not willing to participate.

### Data collection

In this study, data were collected from two primary groups of participants, caregivers, and healthcare workers. For the caregivers, the questions focused on various aspects including socio-demographic information, level of knowledge about SCD and Newborn NBS for SCD, attitudes toward NBS, and their willingness to accept it. The questionnaire also assessed their understanding of SCD as a genetic disorder, familiarity with different hemoglobin genotypes ie Hb AA, Hb AS/AC, Hb SS, Hb SC, and recognition of associated signs and symptoms in SCD. In addition, it explored their sources of information about NBS, willingness to have their babies undergo screening, and acceptance of the screening results. Furthermore, it inquired about their preferences regarding the timing of screening, support for mandatory screening, integration into routine immunization education, and willingness to follow up if the screening indicates a positive result.

For the healthcare workers, the questionnaire assessed their years of experience, familiarity with NBS for SCD, and frequency of recommending it to caregivers. Also, it evaluated their understanding of how NBS for SCD contributes to early detection and management.

The data collected was processed and analyzed using the R statistical software (version 4.3.2).

## Results

### Caregivers

**Figure 2:**
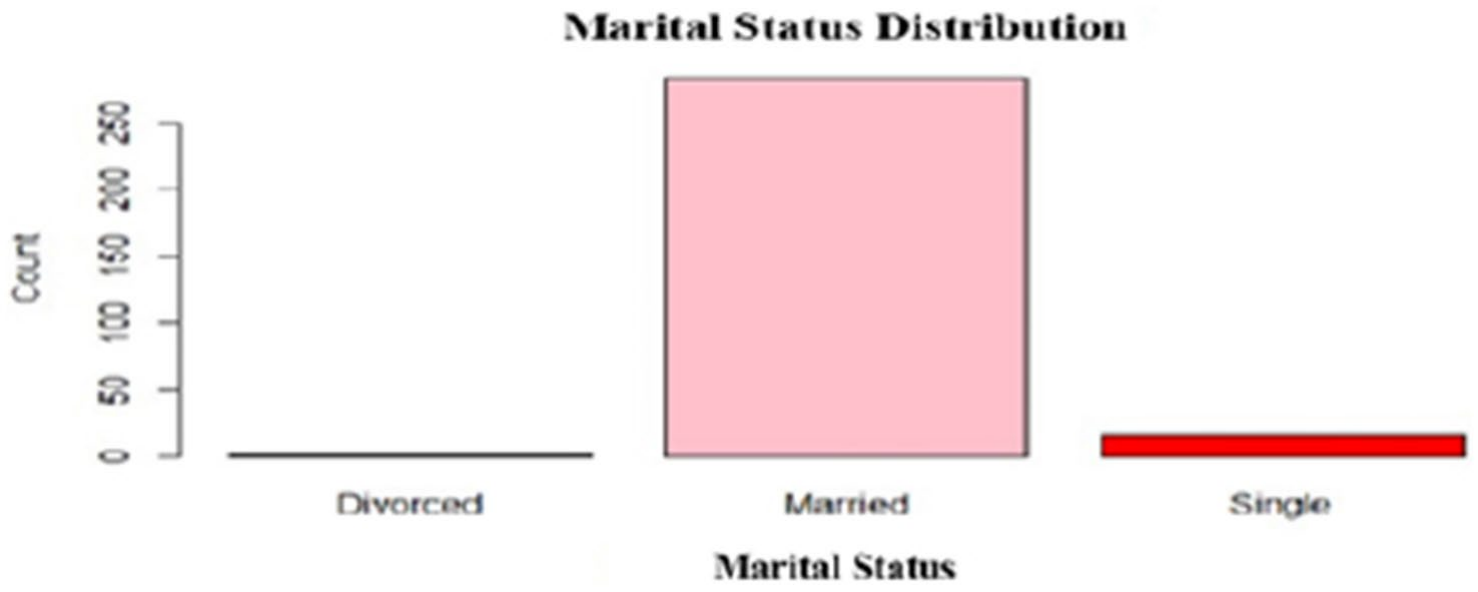
Marital status distribution for caregivers.

A total of 301 caregivers, including both mothers and guardians, participated in the survey. Among them, 283 (94.3%) were married, 16 (5.3%) were single, and 1 (0.3%) was divorced. Regarding education, 109 (36.2%) caregivers had tertiary education, and 163 (54.2%) completed secondary school while 19 (6.3%) had only primary education. Most caregivers were either business owners, 123 (41.1%) or self-employed, 65 (21.7%), with smaller numbers working as civil servants, 37 (12.3%) or unemployed, 58 (19.3%). Of the 301 caregivers, 161 (53.5%) knew their genotype, and a two-third (65.4%) were aware that sickle cell disease is inherited from parents (Table 1; Figure 2).

**Table 1:** Demographic characteristics of caregivers (*n* = 301).

S/No.	Demographic characteristic	Frequency (*n*)	Percentage (%)
1	Marital status		
	Single	16	5.3
	Married	283	94.0
	Divorced	1	0.3
	Missing	1	0.3
2	Age category		
	Young Adults (<=25)	101	33.6
	Adults (26–64 years)	197	65.4
	Older Adults/Seniors (65 years and older)	0	0.0
	Missing	3	1.0
3	Occupation		
	Business	123	40.9
	Civil servant	37	12.3
	Self employed	65	21.6
	Unemployed	58	19.3
	Others	17	5.6
	Missing	1	0.3
4	Religion		
	Christianity	179	59.5
	Islam	119	39.5
	Missing	3	1.0
5	Level of education		
	None	9	3.0
	Primary	19	6.3
	Secondary	163	54.2
	Tertiary	109	36.2
	Others	1	0.3
6	Knowledge of genotype		
	Yes	161	53.5
	No	95	31.6
	Not sure	29	9.6
	Missing	16	5.3

Of the 301 caregivers, 147 (48.8%) and 174 (57.8%) identified bone pain and anemia, respectively, as symptoms of SCD. Our study found that only 128 (42.5%) caregivers have heard about newborn screening (NBS) for SCD, with healthcare workers serving as the primary information source for 90.6% of those informed. There was a strong support for NBS, with 97.6% recognizing its value in early detection and management of SCD. About two-third (64.3%) advocated for mandatory NBS and suggested integrating it into health education talk during immunization and antenatal clinics visits. The majority, 89.4% of caregivers expressed a willingness to accept screening results, and 96% indicated they would follow up if their baby was diagnosed with SCD (Tables 2, 3).

**Table 2:** Care givers’ knowledge and awareness of NBS for SCD (*n* = 301).

S/No	Knowledge	Frequency (*n*)	Percentage (%)
1	SCD is a genetic disorder gotten from parents with sickle cell trait		
	Yes	197	65.4
	No	34	11.3
	Not sure	69	22.9
	Missing	1	0.3
2	Which of these statements best describes the sign and symptom of SCD?		
	Headache	54	17.9%
	Anemia	174	57.8%
	Dactylitis	81	26.9%
	Diarrhea	18	5.9%
	Bone pain	147	48.8%
	Yellowness of the eyes	120	39.8%
3	Have you heard about newborn screening for SCD?		
	Yes	128	42.5
	No	171	56.8
	Missing	2	0.7
4	How long have you known about newborn screening for SCD? (*n* = 124)		
	Less than 1 year	96	77.4
	1–2 years	12	9.7
	3 years	8	6.5
	4 years and above	8	6.5

**Table 3:** Acceptability of NBS for SCD by caregivers (*n* = 301).

S/No	Knowledge	Frequency (*n*)	Percentage (%)
1	Do you agree that newborn screening for SCD could help in early detection and management of SCD?		
	Strongly agreed	175	59.1
	Agreed	114	38.5
	Disagreed	7	2.4
	Strongly disagreed	0	0.0
	Missing	3	1.0
2	Will you be willing to participate in the newborn screening for SCD for your child?		
	Yes	290	98.0
	No	4	1.3
	Not sure	5	1.7
3	Do you agree that newborn screening for SCD should be made mandatory for caregivers?		
	Strongly agreed	193	64.3
	Agreed	79	26.3
	Disagreed	28	9.3
	Strongly disagreed	0	0.0
	Missing	1	0.3

### Healthcare workers

**Figure 3:**
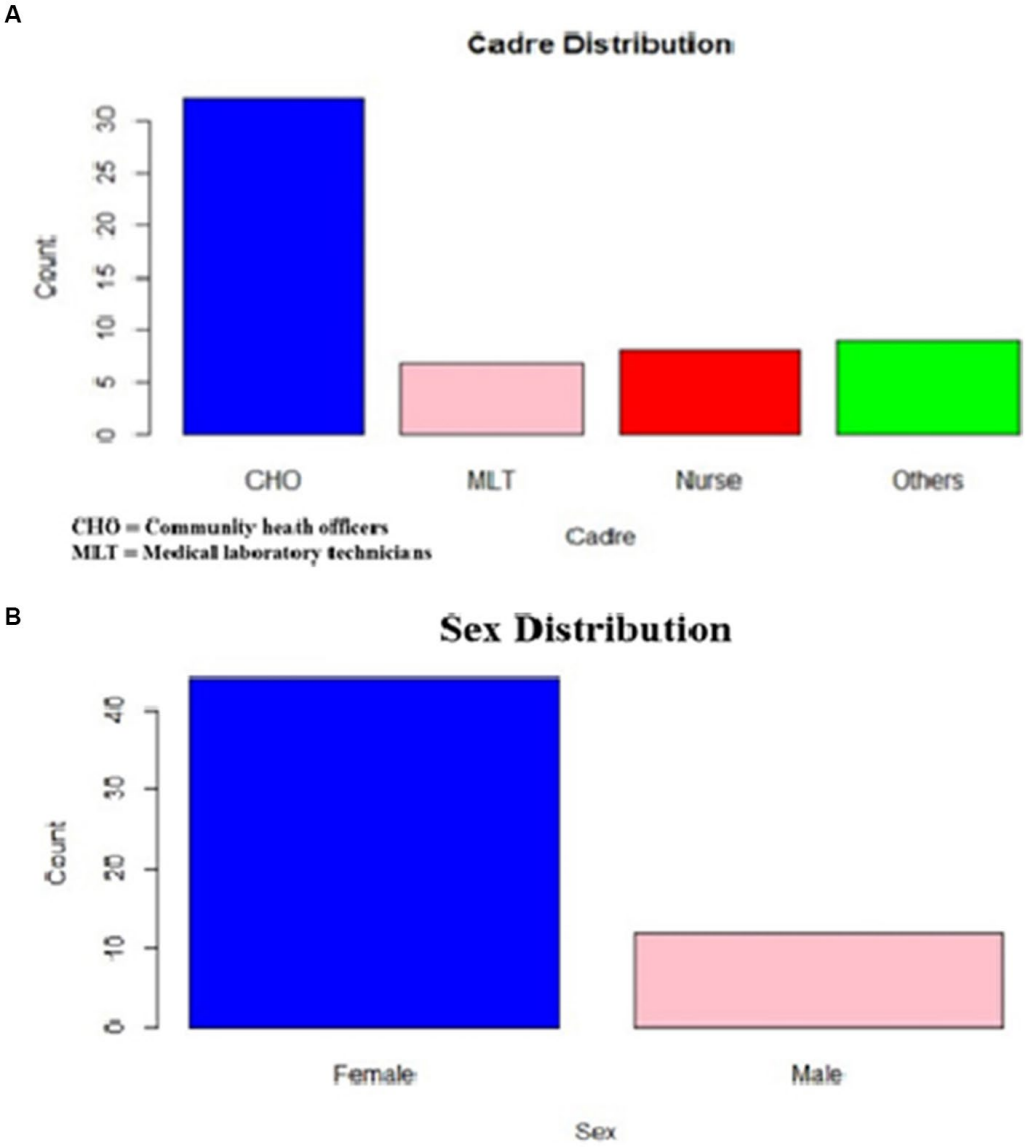
**(A)** Healthcare workers cadre distribution. **(B)** Sex distribution of healthcare worker respondent.

**Figure 4:**
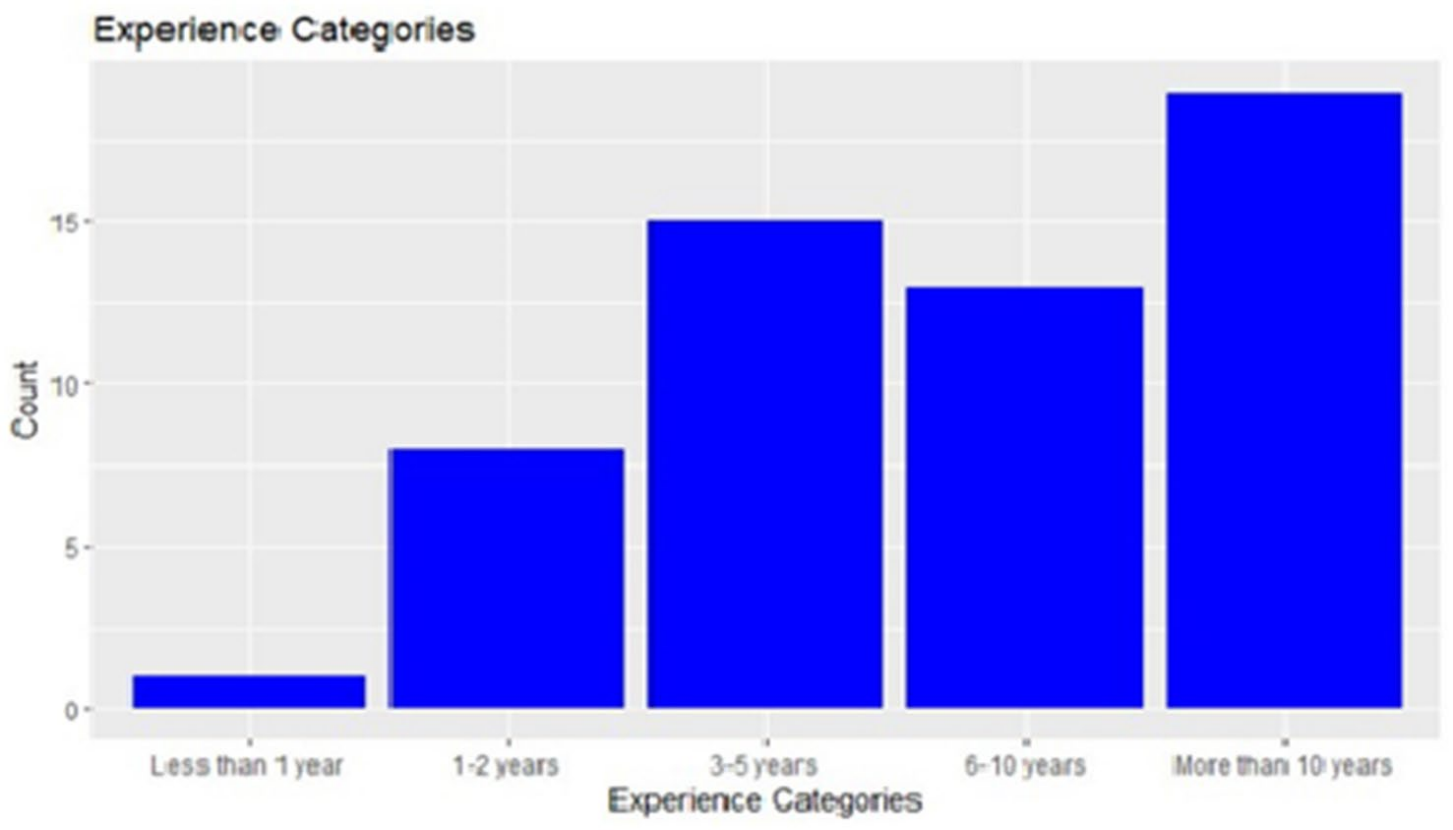
Years of work experience distribution.

**Figure 5:**
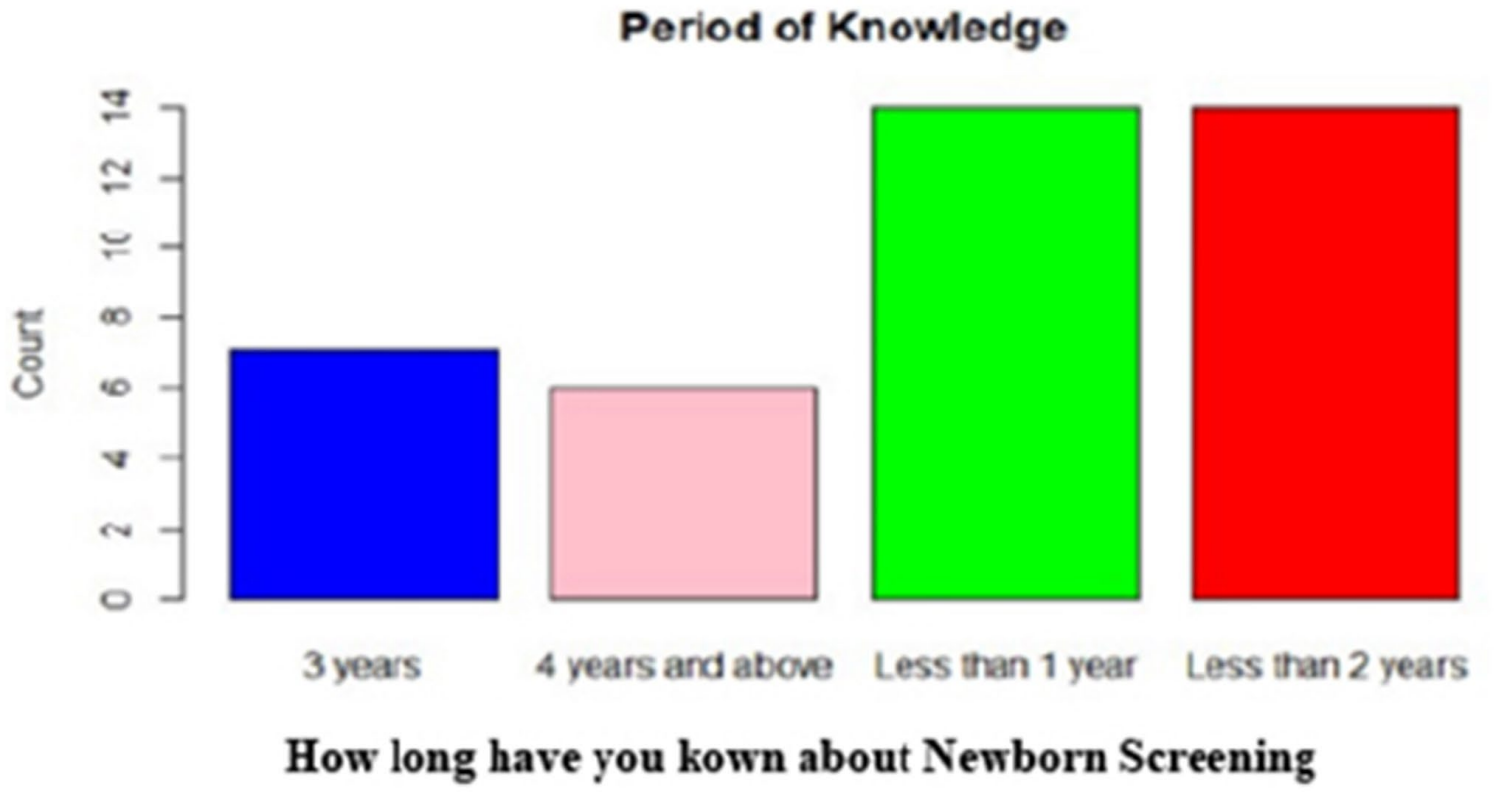
Period of knowledge of NBS among healthcare workers.

A total of 56 healthcare workers, comprising community health officers (CHO) and community health extension workers (CHEWs) medical laboratory technologists and technicians (MLT), nurses, and medical record officers, were interviewed (Figure 3). Half of the healthcare workers (50%) had over 3 years of work experience, with a predominant representation of community health extension workers (57.1%) (Figures 4, 5).

Among the 56 healthcare workers, 39 (73.6%) were aware of newborn screening for SCD, and 30 (72.3%) of them recommended it for caregivers. A majority (80.4%) strongly agreed that newborn screening aids in the early detection and management of SCD. Forty-six, 46 (83.6%) expressed a strong agreement that newborn screening should be mandatory for caregivers and integrated into the health education talk given during immunization and antenatal clinics (87.5%). Concerning the timing of newborn screening, 24 (42.8%) healthcare workers strongly agreed that it should be done within the first 6 weeks of age. However, 15 (26.7%) disagreed, suggesting that allowing babies older than 6 weeks to participate accommodates babies who may miss the initial immunizations. Furthermore, a comparison of healthcare workers’ years of work experience revealed that more experienced individuals were more aware of NBS for SCD, and a similar trend was observed when comparing years of experience to the frequency of recommending newborn screening for SCD (Figure 4).

## Discussion

### Awareness and knowledge about SCD and NBS

This study showed that most care givers had a fair knowledge about SCD as they understood that it is a genetic disease that is inherited from both parents who have sickle cell trait. Also, more than half (53.5%) were aware of their own genotype and were able to identify the commonest symptoms of the disease (Table 2). This is in line with the finding from similar studies within Nigeria (17, 20). However, specifically the level of awareness of NBS for SCD was low consistent with a similar study conducted in Ibadan Western Nigeria which found that less than 50% of the respondent were aware of NBS (18). This may be due to poor public sensitization programs through the mass media which is more effective in reaching the general public than through the health workers from which greater than 90% of the respondents obtained their information. Also, the general lack of routine NBS services in our healthcare facilities may be a contributory factor to this low level of awareness. The level of awareness of NBS among the health care workers was 73.6%, though about a quarter of the respondents indicated that they had not heard about it, these may belong to those cadres that are not directly involved in NBS. All health care workers need to be aware and have adequate knowledge of SCD and NBS because they are an important source of information to be able to bridge the health information gap among the care givers.

### Acceptability and attitude toward NBS for SCD

The health care givers had a very positive attitude toward NBS for SCD though most of them have not heard about it. Almost all (97.6%) respondents in this category believed in the importance of NBS for early detection and management of SCD. They equally believed it should be mandatory and willingly accepted to present their babies for screening (Table 3). This is in keeping with the findings from studies conducted across the various geopolitical regions of the country by Nnodu et al. nation-wide (17), Oluwole and Babalola in the South-West (18, 20), and Nnachi in the South-East (19). These positive attitudes suggest that public educational and sensitization campaigns as well as implementation of NBS programs are likely to be successful and should be leveraged by government and non-governmental organizations toward reducing the burden of SCD in Nigeria. The health care workers in this study generally displayed a very positive attitude toward NBS. Over 80% strongly agreed NBS could help in early detection and management of SCD, should be made mandatory for caregivers and should be included as part of the health education talks given during immunization and antenatal clinic. In addition, over 70% of them had recommended it to the care givers that come to their health facilities. However, the health care workers differed in the time the newborns should be screened (Table 4). About a quarter of them felt that NBS should be extended beyond 6 weeks to accommodate infants that have missed the screening in the immunization period.

**Table 4:** Knowledge and acceptability of NBS for SCD by Healthcare workers (*n* = 56).

S/No	Knowledge and acceptability	Strongly agreed	Agreed	Disagreed	Strongly Disagreed	Missing
1	Do you agree that newborn screening for SCD could help in early detection and management of SCD?	45 (80.3%)	10 (17.9%)	0 (0%)	0 (0%)	1 (1.8%)
2	Do you agree that newborn screening for SCD should be made mandatory for caregivers?	46 (82.1%)	8 (14.3%)	1 (1.8%)	0 (0%)	1 (1.8%)
3	Do you agree that newborn screening should be included as part of the health education during immunization and antenatal clinic?	49 (87.5%)	7 (12.5%)	0 (0%)	0 (0%)	0 (0%)
4	Newborn screening for SCD is preferably done within the first 6 weeks of birth	24 (42.9%)	16 (28.5%)	15 (26.8%)	0 (0%)	1 (1.8%)

### Factors determining awareness, knowledge, and attitude toward NBS for SCD

In this study about half and one-third of the respondents had primary and secondary education, respectively, implying that over 90% are literate (Table 1). This might have contributed to their knowledge and understanding of SCD, their acceptance and positive attitude toward NBS. Education is known to be a major determinant of positive change in behavior and practices, especially regarding health (21, 22). Babalola et al. (18) also found that higher educational level among mothers was one of the major factors associated with increased knowledge and positive attitude toward SCD. Among the health workers, the majority had 6 years and above of work experience which appeared to have been the major factor associated with their awareness and positive attitude toward NBS (Figure 4).

## Conclusion

The study generally revealed good knowledge, positive attitude and high acceptance of SCD and NBS among caregivers and women attending immunization and antenatal clinics, and PHC workers, with strong support for making screening mandatory and integrating it into health education initiatives. Awareness of NBS for SCD was low among care givers and women attending antenatal clinic. The major determinant factors were educational level and long years of work experience among the care givers and health care workers, respectively. There is a need to increase awareness of NBS through mass media campaigns and inclusion of NBS for SCD in the health education talks given to care givers and women at the immunization and antenatal clinics.

## Limitation

Factors impeding newborn screening such as cultural beliefs and social beliefs were not included in the data collection tool and hence were not captured.

## Data Availability

The original contributions presented in the study are included in the article/supplementary material, further inquiries can be directed to the corresponding author.
